# Innovations for Incisional Hernia Prevention

**DOI:** 10.3389/jaws.2022.10945

**Published:** 2022-11-03

**Authors:** Hobart W. Harris

**Affiliations:** Department of Surgery, University of California, San Francisco, San Francisco, CA, United States

**Keywords:** incisional hernia, prevention, invention, tension, fascial closure

## Abstract

Incisional hernias are the most frequent long-term complication of abdominal surgery, resulting in considerable patient morbidity and increased health care costs. These hernias frequently result from excessive tension concentrated at points along the suture line of the abdominal closure. While ample research is focused on developing improved repair materials, the optimal solution to the problem of incisional hernias is prevention. Accordingly, some investigators have postulated that incisional hernias can be prevented by distributing tension more evenly along the fascial closure. Herein we describe two novel and ingenious strategies for the improved distribution of tension when closing abdomens (T-Line^®^ Hernia Mesh and the REBUILD Bioabsorbable™) that were conceived of and developed by surgeons.

Dear Editors,

Incisional hernias are the most frequent long-term complication of abdominal surgery, resulting in considerable patient morbidity and increased health care costs. There are 4–5 million abdominal incisions (laparotomies) performed annually in the United States with hernias resulting after approximately 25% of these procedures (1–3). Importantly, incisional hernias result in severe morbidity beyond the cosmetic deformity of a visible bulge in the anterior abdominal wall, including intestinal obstruction, bowel ischemia, enterocutaneous fistula and significant limits on a patient’s physical activity and gainful employment. Consequently, there are over 400,000 incisional hernia repairs performed each year in the United States making it one of the five most common procedures performed by general surgeons. The increase in US health care costs due to incisional hernia repair is estimated to currently exceed eight billion dollars per year, not including the cost of unemployment benefits for this moderately young patient population. Research and clinical experience indicate that incisional hernias frequently result from excessive tension concentrated at points along the suture line of the abdominal closure. These zones of excessive tension produce focal areas of tissue ischemia, decreased wound healing, and “cheese wiring”—sites of anchor point failure where sutures can tear or pull through myofascial tissue (Figure 1). Suture cheese wiring can occur at 6–14 N/cm, pressures that are routinely exceeded since peak abdominal pressures when coughing, sneezing, or vomiting are often greater than 32 N/cm.

**FIGURE 1:**
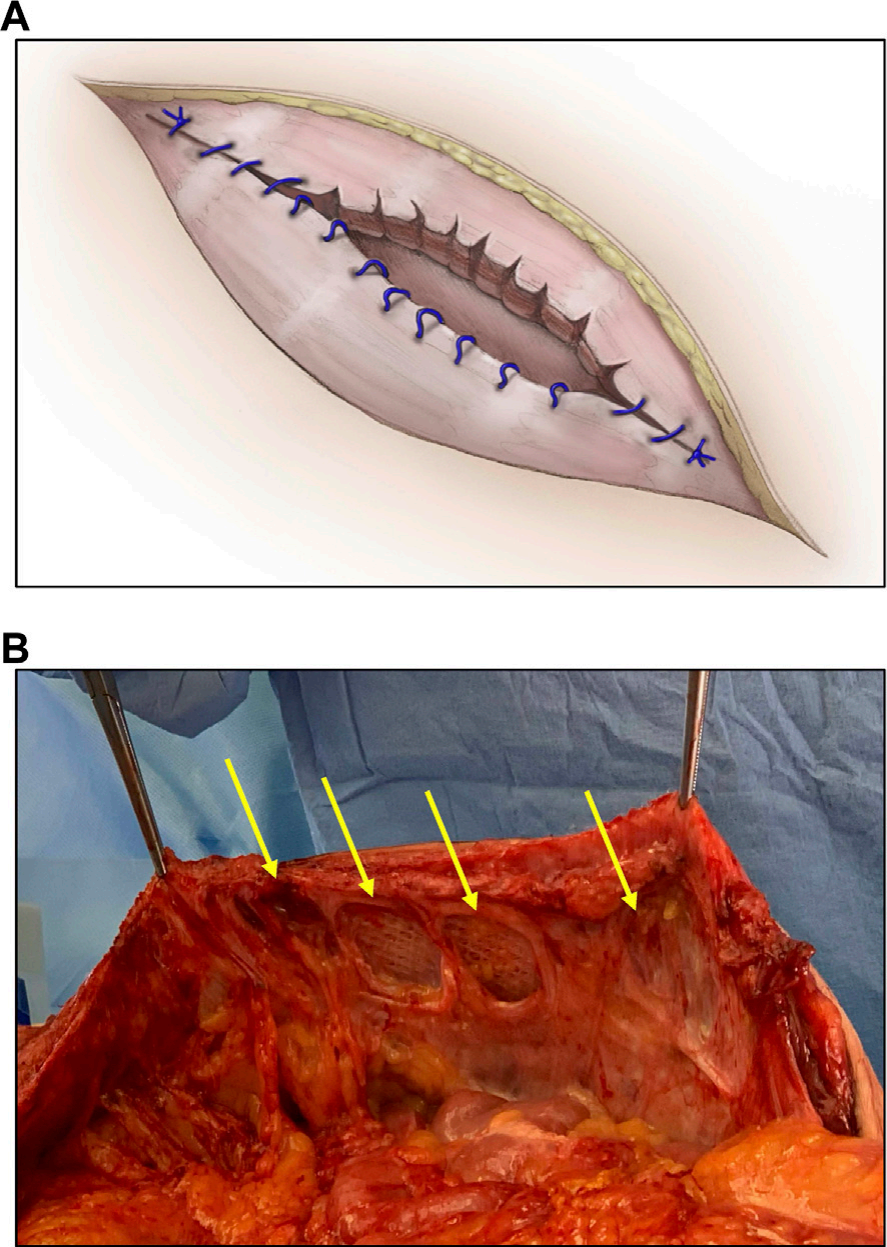
Diagram **(A)** and photograph **(B)** depicting a form of anchor point failure termed cheese wiring wherein sutures tear or cut through tissue at a focal point of attachment and increased tension (arrows).

Despite the magnitude and significance of incisional hernias, research focused on their prevention is sparse. While many studies and current research efforts are focused on improved repair materials, the optimal solution to the problem of incisional hernias is prevention. Notably, some investigators have postulated that incisional hernias can be prevented by more evenly distributing tension along the fascial closure. Support for this simple hypothesis comes from the well-known observation that closing laparotomies using a continuous suturing technique is associated with a decreased incisional hernia rate as compared to an interrupted suture closure (4). Herein we describe two novel and ingenious strategies to distribute tension more evenly when closing abdomens that were conceived of and developed by surgeons. T-Line^®^ Hernia Mesh (Deep Blue Medical Advances, Inc., Durham, NC) and the REBUILD Bioabsorbable™ System (AbSolutions Med, Inc., Mountain View, CA) represent deep insights born of clinical experience as the foundation for unique solutions to a common problem, further highlighting the tradition of the surgeon-inventor.

## T-Line^®^ Hernia Mesh

**FIGURE 2:**
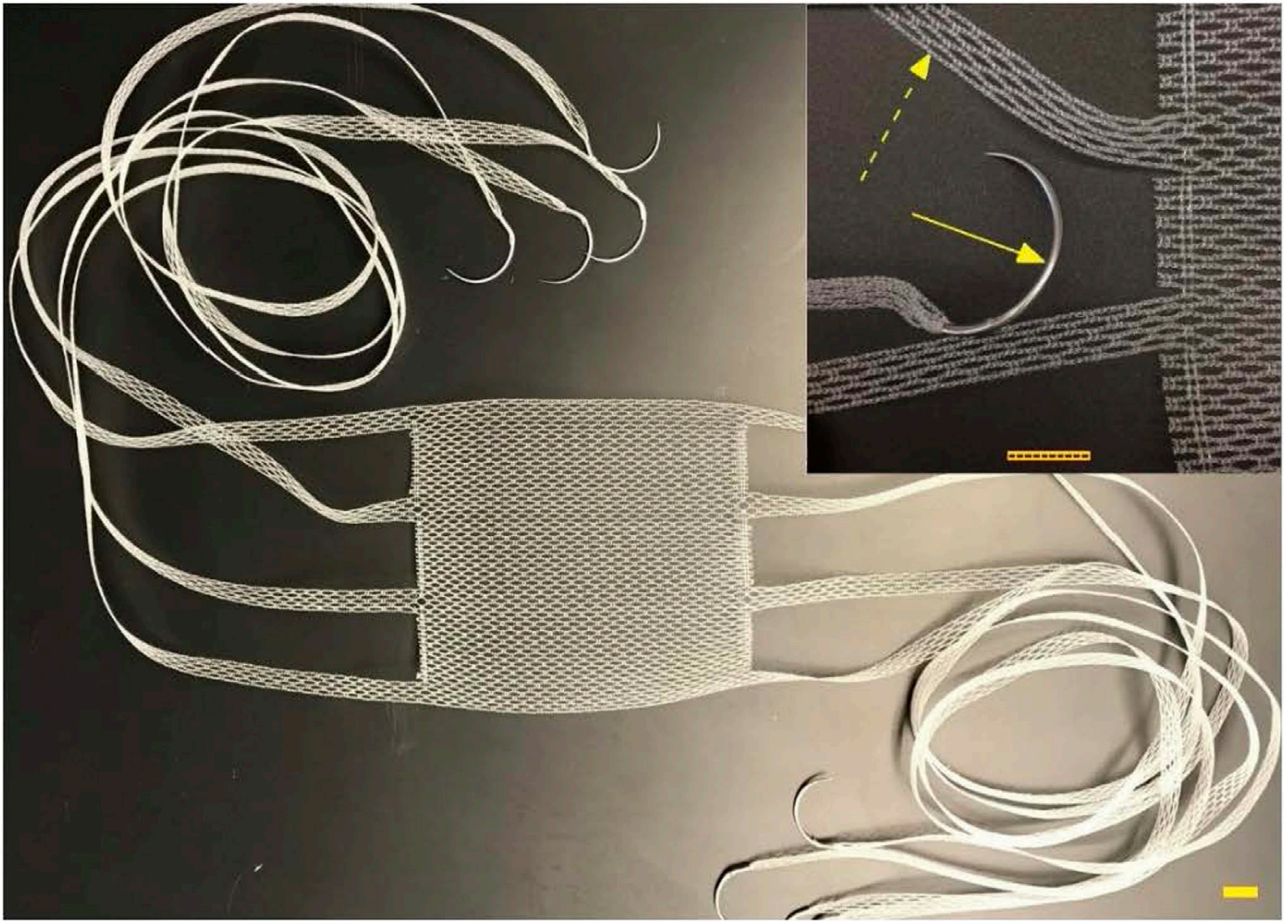
T-line Hernia Mesh: 0.5 cm wide extensions emanating from body of textile with GS21 needles swaged on ends of extensions. Scale bar equals 1 cm; GS-21 needle (solid arrow); integrated mesh extension (dashed arrow). Photo used with permission from Deep Blue Medical Advances, Inc.

**FIGURE 3:**
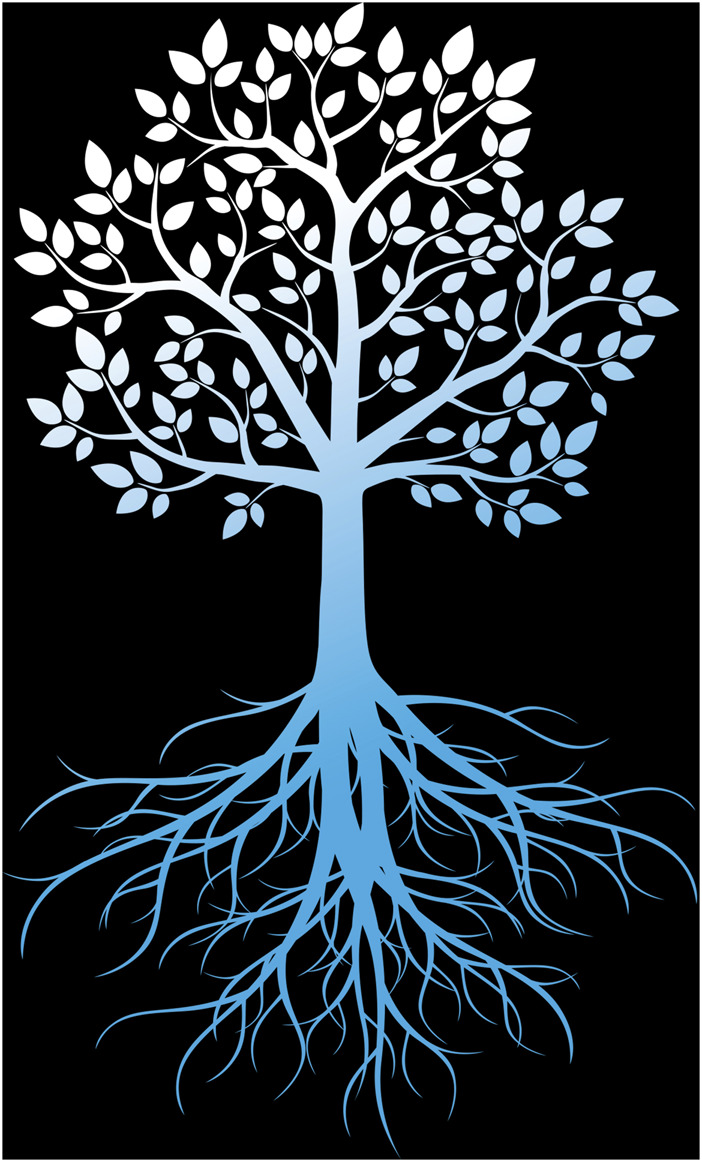
Diagram illustrating the root system that provides anchor strength for the tree.

**FIGURE 4:**
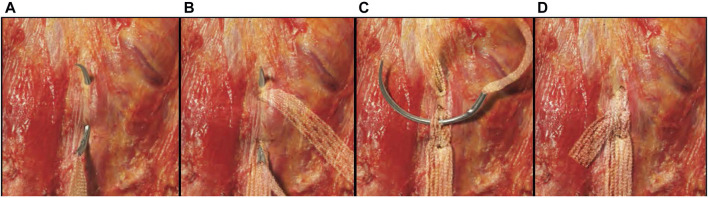
Lock-stitch technique. **(A)** The first bite of the self-locking stitch can be a shallow bite lateral to the edge of the mesh. The extension would then be pulled to create the desired amount of tension on the mesh body. **(B)** The needle is then passed through a center portion of the extension where the first bite entered the fascia and placed slightly deeper through the tissue exiting just lateral to the exit of the first bite. **(C)** The second bite is pulled to create a snug loop around the fascia. The needle is then passed through a center pore of the extension where it exits on the first bite. **(D)** The extension is drawn snug to complete the self-locking stitch, and the excess extension is cut.

T-Line Hernia Mesh is a standard weight (89 g/m^2^), super macroporous (>2.6 mm^2^), polypropylene (prolene) mesh with integrated mesh extensions located at 2-cm intervals along the lateral borders of the prosthetic (Figure 2). Invented by a plastic surgeon, Howard Levinson sought to combine how he was taught to repair tendons in the hand with nature’s strategy for stabilizing tall trees (Figure 3) Similar to the roots of a tree, the T-Line Hernia Mesh extensions increase the surface area across which the prosthetic is anchored. Consequently, the mesh extensions serve to spread the tension and sheer forces over a larger area thereby significantly reducing focal anchor point stress and cheese wiring. While the T-Line Hernia Mesh achieves ∼3-fold stronger anchoring strength than currently available meshes (5), the anchoring strength of the mesh extensions should increase over time as they incorporate with adjacent host tissue. When placed as an onlay, the mesh extensions can be sewn into the adjacent fascia using a quick, self-locking backstitch which secures the extensions and avoids the need for bulky suture knots (Figure 4). Mesh tension is set by sewing the contralateral extensions into tissue, thereby allowing the surgeon to control how tightly the mesh is stretched across the tissue. Notably, the prosthetic has the breaking strength of standard weight prolene mesh, but the handling characteristics of a lightweight mesh due to the specific way in which the mesh fibers are woven together.

An early clinical report involving 18 patients (12 women, mean age 57 years) indicates that the mesh is safe. The surgical site occurrence rate in this high-risk population was favorable with two seromas (11%) and one superficial surgical site infection (6%). While there were no early recurrences, longer follow-up is necessary to determine the product’s effectiveness in terms of hernia prevention and the avoidance of chronic mesh infection.

**FIGURE 5:**
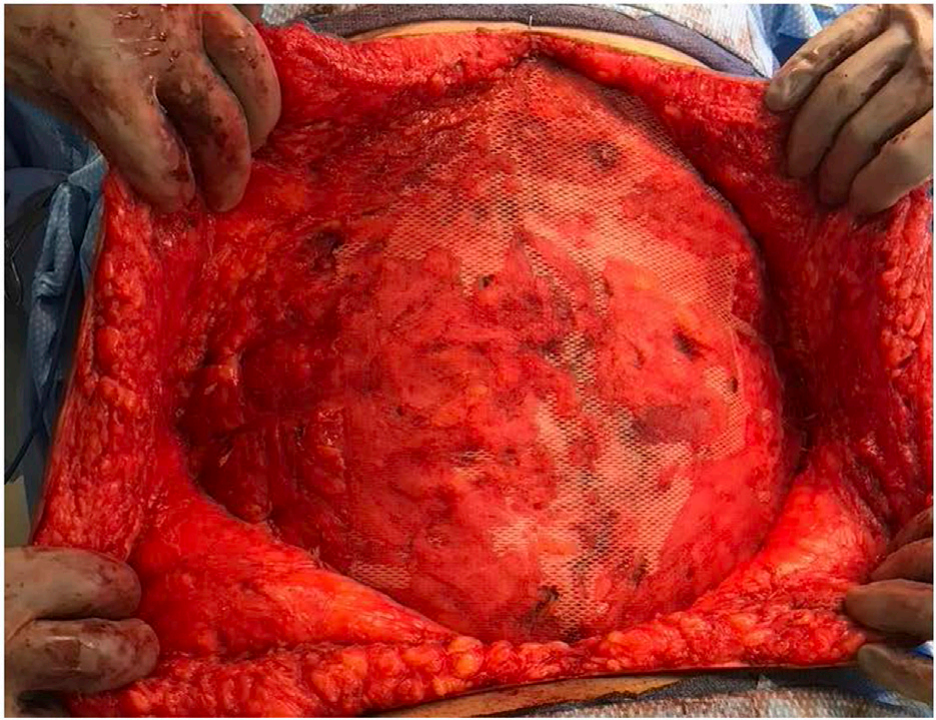
Intraoperative photograph of an onlay mesh repair of a complex ventral hernia using T-Line^®^ Hernia Mesh.

In summary, T-Line^®^ Hernia Mesh translates an observation from nature into a prosthetic design with three important features. First, the integrated mesh extensions effectively eliminate anchor point failure and cheese wiring, two common reasons hernia repairs fail. Second, the macroporous prosthetic material has the tensile strength of standard weight prolene mesh, yet the handling characteristics of a lightweight mesh, which render it easy to use and allow it to readily conform to any variations in the topography of the anterior abdominal wall fascia (Figure 5). Third, the option to remove and reposition the mesh extensions highlights the flexibility of the product, supporting the frequent need for surgeons to be creative when repairing complex ventral hernias. Accordingly, the inventor and Deep Blue Medical Advances, Inc. are expanding the potential applications of this novel technology by introducing a product combined with an adhesion barrier that will be suitable for placement within the peritoneal cavity, plus a biodegradable version for use when looking to avoid placing a permanent mesh.

## REBUILD Bioabsorbable™

**FIGURE 6:**
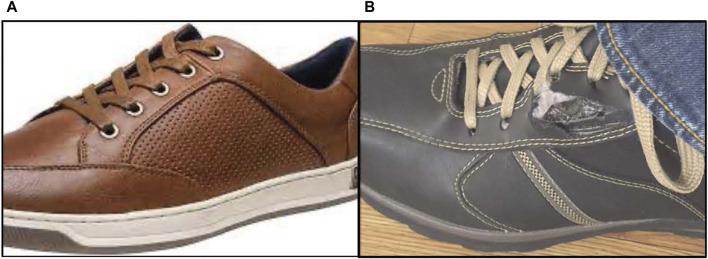
Reinforced eyelets **(A)** prevent shoelaces from tearing through **(B)**.

**FIGURE 7:**
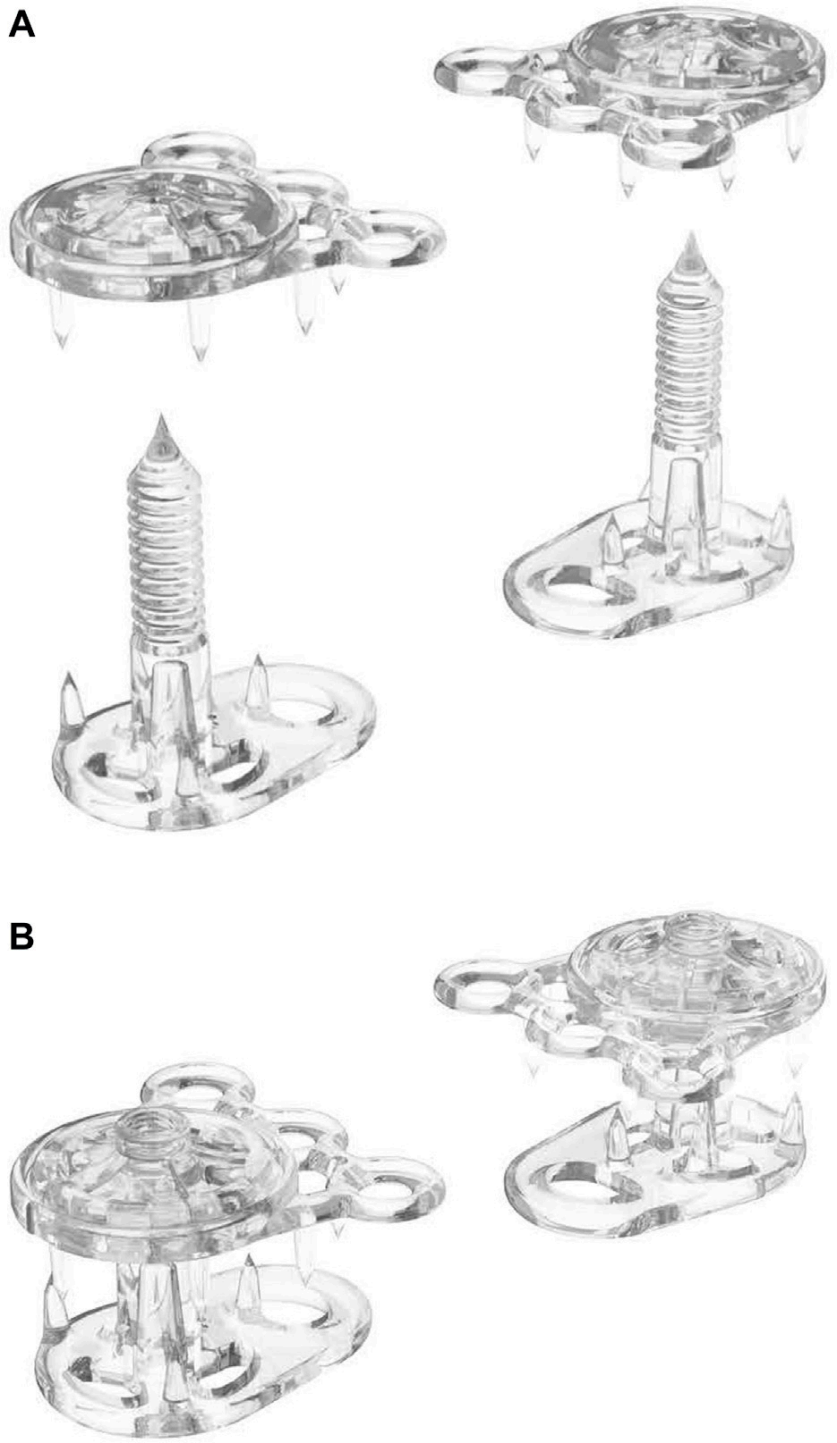
A REBUILD™ pair consisting of two Anterior Plates **(A)** and two Posterior Plates **(B)** before being combined into single anchor units **(B)**.

The REBUILD Bioabsorbable™ is a sterile, single-use implantable device designed for closure of midline abdominal incisions, also co-invented by a plastic surgeon. Dan Jacobs has long been fascinated by the anatomy and function of the anterior abdominal wall, and dubious of traditional teaching around how to best close laparotomy incisions. Convinced that there had to be a better way than conventional suture techniques, Jacobs drew inspiration from how we tie our shoes! Or, more precisely, how reinforced eyelets prevent shoelaces from tearing through the shoe itself (Figure 6). Noting that reinforced eyelets effectively distribute the tension from tightly tied shoelaces, he sought to transfer this simple, yet elegant solution to closure of the abdominal wall. After several design iterations and prototypes, each REBUILD™ unit (think pair of opposing shoe eyelets) consists of two Anterior Tension Distribution (Anterior) Plates and two Posterior Tension Distribution (Posterior) Plates (Figures 7A,B). The Posterior Plate has one prong which is 26 mm tall and three 5.5-mm tines. The Anterior Plate also has five 5.5-mm tines. This suture tension distribution system provides 16-fold the tissue contact area compared to a standard 1 cm by 1 cm, USP #1 running suture closure. The Anterior and Posterior Plates are manufactured from poly-lactide-co-glycolide (PLGA), a biodegradable polymer that is physically strong, highly biocompatible, and whose building blocks are commonly used in suture material (Vicryl). PLGA undergoes bulk degradation by hydrolysis of its ester linkages, resulting in the release of lactate and glycolate which are eliminated from the body after further metabolism.

**FIGURE 8:**
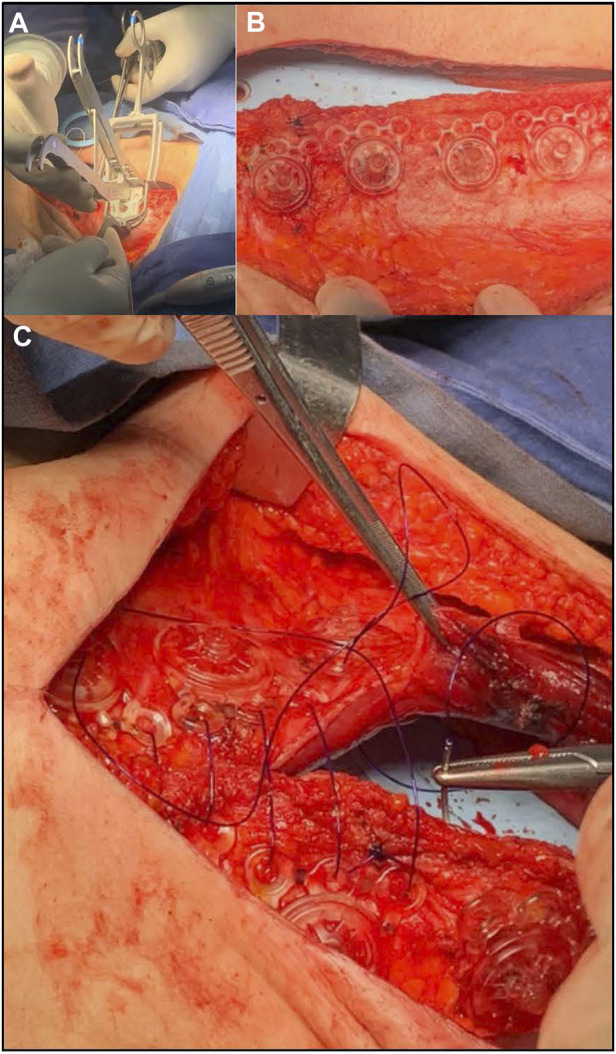
Photographs of REBUILD™ use during surgery. A Posterior Plate with its central soft tissue fixation post is being inserted through the abdominal wall tissue **(A)**, with simultaneous ratchet fixation of an Anterior Plate on post of the Posterior Plate to create a module. A series of these modules are positioned along the midline incision **(B)**, the system is secured with suture placed through the anchor module eyelets, and the excess fixation posts trimmed **(C)**. The REBUILD™ system is provided with deployment tools made from stainless steel **(A)**.

A pair of Posterior Plates, with their central soft tissue fixation posts are inserted through the abdominal wall tissue directly opposite each other across the midline incision (Figure 8A). The Anterior Plates are simultaneously ratcheted to the fixation post of the Posterior Plate to create a single anchor. A series of these anchors are positioned along the midline incision (Figure 8B), the system is secured with suture placed through the device’s eyelets (Figure 8C), and the excess fixation posts are trimmed (Figure 8C).

**FIGURE 9:**
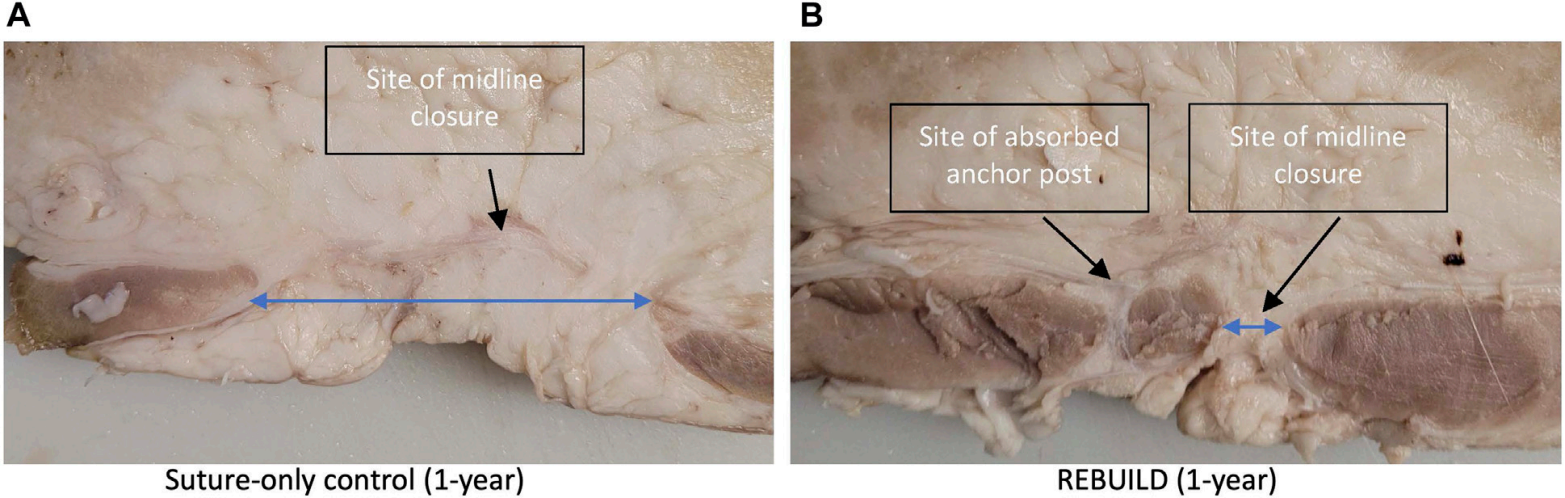
Axial slices of the porcine abdominal walls one year after an animal was closed with standard running suture technique **(A)** compared to an animal closed with REBUILD plus suture **(B)**. Suture-only closure demonstrates a wide gap between the medial borders of the rectus muscles (long blue arrow) versus the narrow gap between the muscles present in the REBUILD-plus-suture closure (short blue arrow). Average gap measurements are 52.6 mm for running suture and 13.5 mm for REBUILD + suture.

**FIGURE 10:**
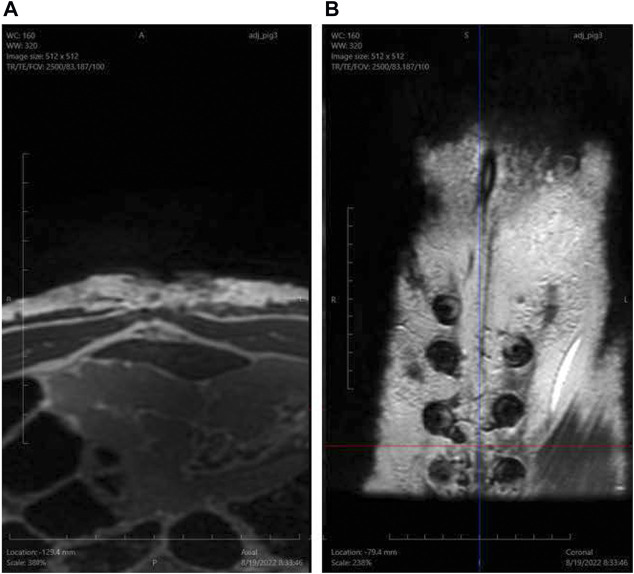
MRI images of the abdomen 37 days after REBUILD-plus-suture closure of the abdominal wall in a porcine model. **(A)** Cross-sectional view demonstrates contiguous rectus muscle without a gap between the medial borders of the rectus muscles. **(B)** Coronal view demonstrates *in vivo* placement of REBUILD Anterior Plates in the subcutaneous (prefascial) plane.

Porcine animal studies were conducted comparing REBUILD to standard suture technique, and although the number of animals is small (two REBUILD test animals and one suture control), the difference in midline integrity at 1 year is dramatic (Figure 9). MRI at 37 days in a separate pig demonstrates in vitro devices in the coronal view and contiguous rectus muscle without a gap at that midline in axial view (Figure 10). While this novel medical device is not FDA approved and thus not yet commercially available, clinical testing is underway with excellent early results.^1^

In summary, T-Line^®^ Hernia Mesh and the REBUILD Bioabsorbable™ leverage simple but effective methods of dispersing force with the goal of mitigating myofascial tissue ischemia and injury, and thus preventing incisional hernias. Whereas the design strategies are very different, both are ingenious translations of common, everyday observations into clinically significant innovative tools that surgeons can use to improve outcomes for patients having abdominal surgery. Furthermore, these devices harken echoes of Theodor Kocher, Alexis Carrel, Michael DeBakey, Patricia Bath, Thomas Fogarty, and numerous other surgeon inventors whose commitment, determination, focus, imagination, and creative spirit benefit us daily.

## Data Availability

The original contributions presented in the study are included in the article/supplementary material, further inquiries can be directed to the corresponding author.

## Footnotes

1 Principal Investigator: Luis Palacios, MD: Surgical Oncology; Instituto de Cancerologia “Las Americas”—AUNA; Medellin, Columbia.
